# A General Strategy for the Stereoselective Synthesis of the Furanosesquiterpenes Structurally Related to Pallescensins 1–2

**DOI:** 10.3390/md17040245

**Published:** 2019-04-25

**Authors:** Stefano Serra

**Affiliations:** Consiglio Nazionale delle Ricerche (C.N.R.) Istituto di Chimica del Riconoscimento Molecolare, Via Mancinelli 7, 20131 Milano, Italy; stefano.serra@cnr.it; Tel.: +39-02-2399 3076

**Keywords:** pallescensin 1, pallescensin 2, dihydropallescensin 2, isomicrocionin-3, pallescensone, furanosesquiterpenes, stereoselective synthesis, lipase-mediated resolution, cyclogeranylsulfonylbenzene isomers

## Abstract

Here, we describe a general stereoselective synthesis of the marine furanosesquiterpenes structurally related to pallescensins 1–2. The stereoisomeric forms of the pallescensin 1, pallescensin 2, and dihydropallescensin 2 were obtained in high chemical and isomeric purity, whereas isomicrocionin-3 was synthesized for the first time. The sesquiterpene framework was built up by means of the coupling of the C_10_ cyclogeranyl moiety with the C_5_ 3-(methylene)furan moiety. The key steps of our synthetic procedure are the stereoselective synthesis of four cyclogeraniol isomers, their conversion into the corresponding cyclogeranylsulfonylbenzene derivatives, their alkylation with 3-(chloromethyl)furan, and the final reductive cleavage of the phenylsulfonyl functional group to afford the whole sesquiterpene framework. The enantioselective synthesis of the α-, 3,4-dehydro-γ- and γ-cyclogeraniol isomers was performed using both a lipase-mediated resolution procedure and different regioselective chemical transformations.

## 1. Introduction

**Figure 1:**
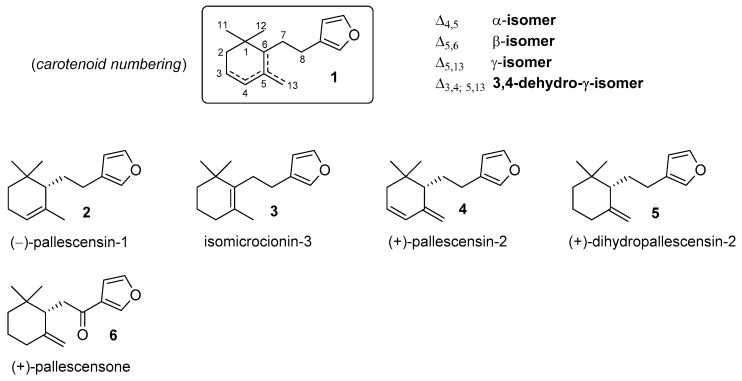
Representative examples of natural furanosesquiterpenes showing molecular framework of type **1**.

The furanosesquiterpenes are a large family of terpenoids that have been isolated from different natural sources. Among these compounds, those possessing a chemical framework consisting of a mono-cyclofarnesyl moiety linked to the 3-furyl moiety (compounds of type **1**, Figure 1) constitute a small subclass whose components occurs only in marine environments. 

The first studies of these natural products date back to 1970s when pallescensin-1 (**2**) and pallescensin-2 (**4**) were isolated from the sponge *Dysidea pallescens* [1], together with other structurally related sesquiterpenes. Afterward, these compounds were also detected in other sponges [2,3] and in some nudibranchs that feed on sponges [4]. Moreover, the pallescensin-1 isomers isomicrocionin-3 (**3**) [3] and dihydropallescensin-2 (**5**) [2,4,5,6,7] were isolated contextually to the above-mentioned studies as well as during the course of researches finalized to the characterization of metabolites derived from marine organisms. In addition, the ketone derivative pallescensone (**6**) was obtained from the dichloromethane extract of the New Zealand sponge *Dictiodendrilla cavernosa* [8], and later, from different nudibranch species [9,10]. 

Almost immediately after their identification, both the chemical structure and the absolute configuration of the pallescensin-1 (**2**) and of the pallescensin-2 (**4**) were confirmed by chemical synthesis [11]. Thereafter, several new synthetic approaches [12,13,14,15,16,17,18,19] provided compounds **2**, **5**, and **6** both in racemic and in enantioenriched forms. Curiously, isomicrocionin-3 (**3**) has not been prepared yet, whereas pallescensis-2 (**4**) has been synthesized only in racemic form.

The reported syntheses were studied in order to confirm a proposed chemical structure or to assign the absolute configuration to a given metabolite. Overall, the preparation of the sesquiterpenes **2**–**6** was studied on a case-by-case basis. Therefore, a reliable and general synthetic approach to this class of compounds is still lacking. In addition, some of these sesquiterpenes have shown biological activity, but the limited amount of the available natural material precluded their comprehensive evaluation. For example, compounds **5** and **6** were isolated from nudibranch mollusks and are thought to possess antifeedant activity against their predators [4,5,6,7,8,9,10]. This ability was experimentally confirmed only on the whole dichloromethane extract of the mollusks [10], thus the determination of the real antifeedant contribute of each furanosesquiterpene could not be determined. Similarly, the antibacterial activity [3] of the pallescensin-1 (**2**) and the inhibitory activity against human tyrosine protein phosphatase 1B (hPTP1B) [7] of the dihydropallescensin-2 (**5**) were evaluated only for the natural occurring (*S*) enantiomers. Since the enantiomeric composition of a given compound could affect its biological properties, it is clear that the reported data are not enough to give a proper characterization of the biological activity of these metabolites. Overall, the aforementioned considerations point to the need of a general and stereoselective synthetic method for the preparation of all the isomeric forms of these furanosesquiterpenes. 

By taking advantage of our previously acquired expertise in the enantioselective synthesis of monocyclofarnesyl terpenoids [20,21,22,23] and cyclogeraniol isomers [24], we decided to devise a synthetic procedure that could comply with the above-described requirements. 

**Figure 2:**
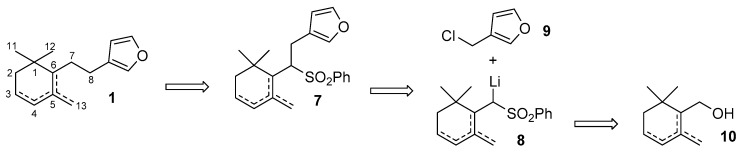
The proposed retrosynthetic analysis for the stereoselective synthesis of the furanosesquiterpenes possessing molecular framework of type **1**.

Our synthetic plan is exemplified by the retrosynthetic analysis described in Figure 2. Accordingly, we envisioned to prepare the target molecules of type **1** through the reductive cleavage of the phenylsulfonyl derivatives of type **7**, which can be in turn obtained by alkylation of the cyclogeranylsulfonylbenzene derivatives **8** with 3-(chloromethyl)furan **9**. The latter lithium salt can be prepared by reaction of a commercially available alkyllithium reagent (e.g., BuLi) with the cyclogeranylsulfonylbenzene derivatives, in turn synthesizable from the corresponding enantiopure cyclogeraniol isomers **10**. Of course, none of the above-described chemical transformations should involve side reactions, such as the double bond isomerization or the racemization, which could end up with decreasing the isomeric purity of the chemical intermediates, and thus, of the target compounds **1**.

Herein, we describe the accomplishment of this synthetic plan, whose effectiveness was confirmed by the stereoselective preparation of some selected furanosesquiterpenes, namely (*S*)-pallescensin-1 (−)-**2**, isomicrocionin-3 (**3**), (*R*)-pallescensin-2 (−)-(**4**), and (*R*)-dihydropallescensis-2 (−)-(**5**). The limits of the presented approach were also discussed as highlighted with the stereoselective synthesis of (*R*)-pallescensone (−)-(**6**), which again required a building block of type **10** as starting material but with the use of a different synthetic path.

## 2. Results and Discussion

**Figure 3:**
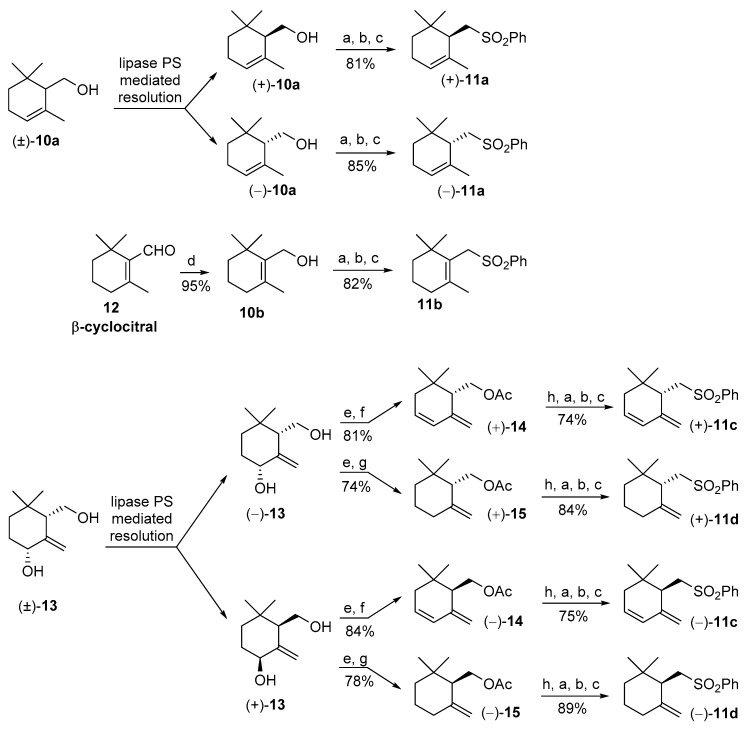
Synthesis of the stereoisomeric forms of the cyclogeranylsulfonylbenzene derivatives **11a-d** starting from the racemic cyclogeraniol derivatives **10a** and **13** and from β-cyclocitral **12**. Reagents and conditions: (**a**) TsCl, Py, CH_2_Cl_2_, DMAP catalyst, rt (room temperature), 4 h; (**b**) K_2_CO_3_, DMSO, PhSH, rt, 12 h; (**c**) H_2_O_2_, MeOH, (NH_4_)_2_MoO_4_ catalyst, 0 °C then rt, 8 h; (**d**) NaBH_4_, MeOH, 0 °C; (**e**) Ac_2_O, Py, DMAP catalyst, rt, 8 h; (**f**) CaCO_3_, PPh_3_, Pd(OAc)_2_ catalyst, dioxane, reflux, 5 h; (**g**) HCOOH, Et_3_N, (PPh_3_)_2_PdCl_2_ catalyst, PPh_3_, THF, reflux, 6 h; (**h**) NaOH, MeOH, reflux.

According to our retrosynthetic analysis, we started with the stereoselective preparation of the cyclogeraniol isomers **10**. The α-cyclogeraniol enantiomers were already prepared using both asymmetric synthesis [25] and resolution procedure [26]. Since racemic α-cyclogeraniol is easily synthesizable from ethyl geraniate [24], the lipase-mediated resolution procedure is the most suitable approach for the preparation of both the enantiomeric forms (Figure 3) of the alcohol **10a**. 

In order to find out a proper enzyme to be employed in this process, we investigated the reactivity of (±)-**10a** toward irreversible acetylation using vinyl acetate as acyl donor in the presence of lipase catalyst. We checked three different commercial enzymes, namely porcine pancreatic lipase (PPL), *Candida rugosa* lipase (CRL), and lipase from *Pseudomonas* sp., (lipase PS). These preliminary experiments indicated that only lipase PS catalyzed the esterification reaction with an enantioselectivity acceptable to perform a proper enantiomers separation (enantiomeric ratio E = 9.2). Our findings agree with previous reported studies on the same enzymatic transformation [26], which assessed an enantiomeric ratio of 12.9 for lipase PS. 

Accordingly, our resolution procedure furnished the enantioenriched alcohols (*S*)-(+)-**10a** and (*R*)-(−)-**10a** that were converted in the corresponding sulfones (*S*)-(+)-**11a** and (*R*)-(−)-**11a**, respectively. This chemical transformation was accomplished by means of a high yielding, three steps procedure [27], consisting of the reaction of alcohol **10a** with tosyl chloride, nucleophile substitution of the obtained tosylate with potassium thiophenate in dry DMF, followed by sodium molybdate catalysed oxidation of the resulting sulfide using an excess of hydrogen peroxide in methanol. Other synthetic methods, usually employed for the transformation of a hydroxyl functional group into a phenylsulfonyl group, afford sulfones **11** in inferior yields. For example, the reaction of the diphenyldisulfide/tributylphosphine reagent [28] with 3,4-dehydro-γ-cyclogeraniol or with γ-cyclogeraniol give the expected sulfide derivatives close to a significant amount of elimination side products. For this reason, we decided to employ exclusively the above-described thiophenate displacement procedure for the synthesis of the four sulfones **11a**–**d**.

Concerning β-cyclogeraniol, we selected β-cyclocitral **12** as starting compound. The latter aldehyde is commercially available since it is employed both as building block for carotenoids synthesis and as flavor ingredient [29,30]. The reduction of **12** with NaBH_4_ in methanol afforded quantitatively β-cyclogeraniol **10b** that was converted into sulfone **11b** according to the general procedure described above.

For the stereoselective synthesis of γ-isomers **11c** and **11d**, we used diol **13** as a common starting compound. According to our previous studies [24], the latter racemic diol is preparable in high diastereoisomeric purity starting from α-cyclogeraniol. The following lipase PS mediated resolution procedure afforded diols (4*R*,6*S*)-(−)-**13** and (4*S*,6*R*)-(+)-**13** in high enantiomeric purity. Each one of these two enantiomers was transformed into the corresponding enantiomeric forms of the acetates **14** and **15**. Accordingly, the chemical acetylation of the diol **13** enantiomers afforded the corresponding diacetates that were submitted to two different chemical reactions, aimed to the cleavage of the secondary allylic acetate group. The regioselective elimination of the latter group was accomplished refluxing the diacetates in dioxane, in presence of calcium carbonate and palladium acetate catalyst [31]. This process allow the conversion of the diols (−)-**13** and (+)-**13** into diene derivatives (+)-**14** and (−)-**14**, respectively. Similarly, the diacetate derivatives of the diols (−)-**13** and (+)-**13** were reduced using triethylammonium formate, in refluxing tetrahydrofuran (THF) and in presence of the palladium catalyst to afford γ-cyclogeraniol acetate enantiomers (+)-**15** and (−)-**15**, respectively [24]. It is worth noting that both reactions proceeded without appreciable formation of other isomers deriving from double bonds isomerization.

This aspect is of pivotal relevance in natural product synthesis, where the biological activity of a given product is often dependent on its isomeric composition. Finally, the obtained acetates enantiomers (+)- and (−)-**14**, (+)- and (−)-**15** were hydrolyzed using sodium hydroxide in methanol, and the obtained alcohols were transformed into sulfones (+)- and (−)-**11c**, (+)- and (−)-**11d**, respectively, according to the general procedure used for the synthesis of compounds **11a** and **11b**.

The obtained sulfones were then used as chiral building blocks for the stereoselective synthesis of the marine furanosesquiterpenes structurally related to pallescensins. Although we prepared both enantiomers of the compounds **11a**, **11c**, and **11d**, the isomers (−)-**11a**, (−)-**11c**, and (−)-**11d** were those available in higher enantiomeric purity, according to the resolution procedures of alcohol **10a** and diol **13**. Therefore, we decided to use the latter sulfone enantiomers for the furanosesquiterpenes synthesis. 

**Figure 4:**
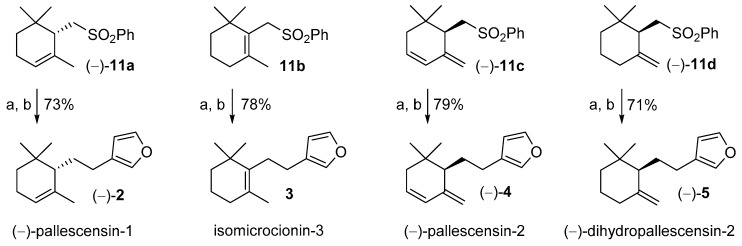
Use of the cyclogeranylsulfonylbenzene derivatives **11a**–**d** in the stereoselective synthesis of the furanosesquiterpenes (−)-pallescensin-1 (**2**), isomicrocionin-3 (**3**), (−)-pallescensin-2 (**4**), and (−)-dihydropallescensis-2 (**5**). Reagents and conditions: (**a**) *n*BuLi, THF dry, −60 °C, then **9** in DMPU, rt, 2 h; (**b**) lithium naphthalenide, THF dry, Et_2_NH, −75 °C, 1 h.

As described in the retrosynthetic analysis, compounds (−)-**11a**, **11b**, (−)-**11c**, and (−)-**11d** were treated with *n*-butyllithium (*n*BuLi) and the resulting lithium salts were alkylated using 3-(chloromethyl)furan **9**. The obtained derivatives **7a-d** were not isolated and were treated with lithium naphthalenide at low temperature (−70 °C) in order to remove the phenylsulfonyl group through its regioselective reduction. Both alkylation step and phenylsulfonyl group cleavage proceeded with high chemical yields and compounds (−)-**11a**, **11b**, (−)-**11c**, and (−)-**11d** were efficiently and stereoselectively converted into (−)-pallescensin-1 (**2**), isomicrocionin-3 (**3**), (−)-pallescensin-2 (**4**), and (−)-dihydropallescensin-2 (**5**), respectively (Figure 4). 

The proposed synthesis of compounds **2**, **4**, and **5** compare favorably over the previously reported stereoselective methods [11,15,18,19] since the overall yields are higher, the approach is operationally simple, and it afforded the target compounds in high stereoisomeric purity.

Isomicrocionin-3 was not synthesized before. Therefore, the comparison of the analytical data of synthetic **3** with those recorded for the natural sesquiterpene isolated from *Fasciospongia* sp. [3] allows us confirming the chemical structure previously assigned to isomicrocionin-3.

Furthermore, as mentioned in the introduction, pallescensis-2 (**4**) was synthesized only in racemic form [11] and the (*S*) absolute configuration was tentatively assigned to the dextrorotatory isomer. This assumption is based on the observation that both (+)-pallescensin-2 and (−)-pallescensin-1 were isolated from the same sponge (*Dysidea pallescens*) and the absolute configuration of (−)-pallescensin-1 was already assigned by chemical correlation with (*S*)-(−)-α-cyclocitral. (*S*)-(−)-**2** and (+)-**4** most likely possess the same absolute configuration because they were formed through a common biosynthetic pathway. According to our synthetic procedure, we established a chemical correlation between (4*S*,6*R*)-4-hydroxy-γ-cyclogeraniol (+)-**13** and (−)-pallescensin-2 (**4**), thus confirming unambiguously that (−)-pallescensin-2 (**4**) possesses (*R*) absolute configuration.

The very good results described above prompted us to investigate a possible exploitation of the sulfone alkylation approach for the synthesis of pallescensone (**6**), a sesquiterpene ketone structurally related to dihydropallescensin-2 (**5**). As described previously [27,32], the lithium salt of a given phenylsulfonyl derivative can be acylated using anhydrous magnesium bromide and the alkyl ester of the corresponding acyl moiety. The following cleavage of the phenylsulfonyl group by means of lithium naphthalenide at low temperature provides the acylated derivative. Unfortunately, we found that the reaction of phenylsulfone **11d** with 3-furoic acid methyl ester afforded the acylated sulfone in very low yield. 

This disappointing result is most likely due to the steric hindrance around the new formed bond, which does not allow phenylsulfonyl and ketone functional groups to adopt vicinal conformation with formation of the magnesium complex, whose chemical stability secure the product formation. 

**Figure 5:**
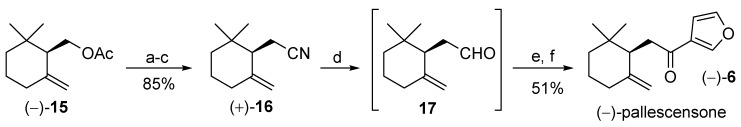
Stereoselective synthesis of (−)-pallescensone (**6**) starting from γ-cyclogeraniol acetate (−)-**15**. Reagents and conditions: (**a**) NaOH, MeOH, reflux; (**b**) TsCl, Py, CH_2_Cl_2_, DMAP catalyst, rt, 4 h; (**c**) NaCN, DMSO dry, 80–90 °C, 5 h; (**d**) DIBAL, toluene, −70 °C, 30 min; (**e**) 3-furyllithium, −70 °C, THF dry, 20 min; (**f**) IBX, DMSO dry, 40 °C, 4 h.

For that reason, we decided to study a different approach for the stereoselective synthesis of ketone **6**. Taking advantage of the above-described process for the preparation of the enantioenriched γ-cyclogeraniol derivatives (Figure 3), we selected the enantiomeric forms of compound **15** as chiral building blocks for pallescensone synthesis. More enantiopure isomer (−)-**15** was used as starting compound (Figure 5) and the devised synthetic procedure provided pallescensone (**6**) after six steps, in good overall yield (43%).

Accordingly, acetate (−)-**15** was hydrolyzed using sodium hydroxide in methanol and the obtained γ-cyclogeraniol was treated with tosyl chloride and pyridine, in presence of the DMAP catalyst. The nucleophilic substitution of the tosyl functional group with the cyanide group was performed by reaction with sodium cyanide in dimethylsulfoxide (DMSO), heating at 80–90 °C to afford cyanide (+)-**16** in 85% overall yield. The latter compound was then reduced at low temperature (−70 °C) using DIBAL in toluene. The resulting aldehyde **17** was not purified and was treated with freshly prepared 3-furyllithium in THF. The obtained crude carbinol was dissolved in dry DMSO and was treated with an excess of IBX [33] to afford (−)-(*R*)-pallescensone (**6**) in 51% overall yield from **16**. 

The ^1^H- and ^13^C-NMR spectroscopic data of the synthesized compound **6** were superimposable with those reported for the synthetic [18,19] and the natural [8] sesquiterpene, whereas the measured optical rotation value, [α]^20^_D_ = −34.4 (*c* 1.1, CH_2_Cl_2_), show comparable value and opposite sign of the naturally occurring (*S*)-pallescensone, [α]^20^_D_ = +36 (*c* 1.0, CHCl_3_).

Finally, it should be considered that the enantiomeric forms of γ-homocyclogeranial **17** were used as a chiral building blocks not only for the synthesis of pallescensone, but also for the preparation of other sesquiterpenes [18,34] or sesquiterpene analogues [35], thus expanding the prospective utility of this synthon in natural products synthesis.

## 3. Materials and Methods

### 3.1. Materials and General Methods

All moisture- and air-sensitive reactions were carried out using dry solvents under a static atmosphere of nitrogen.

All solvents and reagents were of commercial quality and were purchased from Sigma-Aldrich (St. Louis, MO, USA) with the exception of β-cyclogeraniol, 3-(chloromethyl)furan and IBX. β-Cyclogeraniol was prepared by reduction of β-cyclogeranial using NaBH_4_ in methanol. 3-(Chloromethyl)furan was obtained starting from furan-3-carboxylic acid by means of reduction with LiAlH_4_ and reaction of the obtained carbinol with mesyl chloride in presence of *s*-collidine and LiCl [36,37]. IBX was prepared starting from *o*-iodobenzoic acid, according to the literature [33]. 

Lipase from *Pseudomonas cepacia* (PS), 30 units/mg, was purchased from Amano Pharmaceuticals Co., Tokyo, Japan. Enantioenriched α-cylogeraniol **10a** and *cis*-4-hydroxy-γ-cyclogeraniol **13** were prepared by means of the lipase PS-mediated resolution of the corresponding racemic compounds, as previously described by Vidari [26] and Serra [24], respectively. 

### 3.2. Analytical Methods and Characterization of the Chemical Compounds

^1^H and ^13^C-NMR spectra and DEPT (Distortionless enhancement by polarization transfer) experiments: CDCl_3_ solutions at room temperature using a Bruker-AC-400 spectrometer (Billerica, MA, USA) at 400, 100, and 100 MHz, respectively; ^13^C spectra are proton-decoupled; chemical shifts in ppm relative to internal SiMe_4_ (0 ppm).

Thin-layer chromatography (TLC) involved the use of Merck silica gel 60 F_254_ plates (Merck Millipore, Milan, Italy), while column chromatography involved the use of silica gel. 

Melting points were measured on a Reichert apparatus equipped with a Reichert microscope and are uncorrected.

Optical rotations were measured on a Jasco-DIP-181 digital polarimeter (Jasco, Tokyo, Japan).

Mass spectra were recorded on a Bruker ESQUIRE 3000 PLUS spectrometer (ESI detector, Billerica, MA, USA) or by GC-MS analyses.

GC-MS analyses involved the use of an HP-6890 gas chromatograph equipped with a 5973 mass detector, using an HP-5MS column (30 m × 0.25 mm, 0.25-μm film thickness; Hewlett Packard, Palo Alto, CA, USA) with the following temperature program: 60° (1 min), then 6°/min to 150° (held at 1 min), then 12°/min to 280° (held 5 min); carrier gas: He; constant flow 1 mL/min; split ratio: 1/30; *t*_R_ given in minutes.

The values of *t*_R_ for each compound are as follows: *t*_R_(**2**) 18.70, *t*_R_(**3**) 19.14, *t*_R_(**4**) 18.47, *t*_R_(**5**) 18.48, *t*_R_(**6**) 21.81, *t*_R_(**9**) 3.90, *t*_R_(**10a**) 10.59, *t*_R_(**10b**) 11.32, *t*_R_(**11a**) 25.85, *t*_R_(**11b**) 26.03, *t*_R_(**11c**) 25.71, *t*_R_(**11d**) 25.86, *t*_R_(**14**) 14.02, *t*_R_(**15**) 13.86, *t*_R_(**16**) 14.71. 

### 3.3. Stereoselective Preparation of (R) and (S) Enantiomers of 3,4-Dehydro-γ-cyclogeraniol Acetate **14** and γ-Cyclogeraniol Acetate **15**

#### 3.3.1. (*R*)-3,4-Dehydro-γ-cyclogeraniol Acetate (−)-**14**

A sample of diol (+)-**13**, (0.5 g, 2.9 mmol; [α]^20^_D_ = +29.5 (*c* 2.8, CHCl_3_); 98% ee by chiral GC) was converted to the corresponding diacetate by treatment with pyridine (10 mL), DMAP (50 mg, 0.4 mmol) and Ac_2_O (10 mL) at rt for 8 h. After removal of the solvents, the crude diacetate was dissolved in dioxane (20 mL) and treated under N_2_ with Pd(OAc)_2_ (50 mg, 0.2 mmol), CaCO_3_ (1 g, 10 mmol), and PPh_3_ (270 mg, 1 mmol). The resulting heterogeneous mixture was stirred under reflux for 5 h (TLC monitoring). The mixture was then cooled to room temperature, diluted with diethyl ether (100 mL), and filtered. The filtrate was washed successively with saturated aqueous NaHCO_3_ solution (50 mL) and brine, dried (Na_2_SO_4_), and evaporated. The residue was purified by chromatography (*n*-hexane/Et_2_O 95:5–8:2) and bulb-to-bulb distillation to give pure 3,4-dehydro-γ-cyclogeraniol acetate (−)-**14** = (*R*)-(6,6-dimethyl-2-methylenecyclohex-3-enyl)methyl acetate (480 mg, 84% yield) as a colourless oil; [α]^20^_D_ = −69.9 (*c* 3.3, CHCl_3_); 99% of chemical purity by GC. ^1^H NMR (400 MHz, CDCl_3_) δ 6.08 (d, J = 9.8 Hz, 1H), 5.70-5.63 (m, 1H), 4.95 (s, 1H), 4.86 (s, 1H), 4.23 (dd, J = 10.7, 4.6 Hz, 1H), 3.93 (dd, J = 10.7, 9.1 Hz, 1H), 2.21 (dd, J = 9.1, 4.6 Hz, 1H), 2.05 (d, J = 18.6 Hz, 1H), 2.02 (s, 3H), 1.83 (dd, J = 18.6, 5.2 Hz, 1H), 1.02 (s, 3H), 0.92 (s, 3H). ^13^C NMR (100 MHz, CDCl_3_) δ 171.0 (C), 143.3 (C), 127.5 (CH), 127.3 (CH), 114.2 (CH_2_), 64.2 (CH_2_), 50.0 (CH), 37.0 (CH_2_), 31.8 (C), 28.4 (Me), 27.3 (Me), 21.0 (Me). GC-MS *m*/*z* (rel intensity) 134 ([M − AcOH]^+^, 52), 119 (100), 105 (24), 91 (54), 77 (17), 65 (6), 53 (5).

#### 3.3.2. (*S*)-3,4-Dehydro-γ-cyclogeraniol Acetate (+)-**14**

The reaction sequence described above was repeated using sample of diol (−)-**13**, ([α]^20^_D_ = −26.8 (*c* 2.5, CHCl_3_); 90% ee by chiral GC) to afford pure 3,4-dehydro-γ-cyclogeraniol acetate (+)-**14** = (*S*)-(6,6-dimethyl-2-methylenecyclohex-3-enyl)methyl acetate (81% yield) as a colorless oil; [α]^20^_D_ = +61.1 (*c* 3.0, CHCl_3_); 96% of chemical purity by GC. ^1^H-NMR, ^13^C-NMR and GC-MS superimposable to those described for (*R*)-isomer.

#### 3.3.3. (*R*)-γ-Cyclogeraniol Acetate (−)-**15**

A sample of diol (+)-**13**, (0.9 g, 5.3 mmol; [α]^20^_D_ = +29.5 (*c* 2.8, CHCl_3_); 98% ee by chiral GC) was treated with pyridine (10 mL), DMAP (50 mg, 0.4 mmol) and Ac_2_O (10 mL) and set aside at rt until acetylation was complete (8 h). The obtained diacetate was dissolved in dry THF (30 mL) and refluxed under a static nitrogen atmosphere in the presence of formic acid (0.75 g, 16.3 mmol), Et_3_N (1.65 g, 16.3 mmol), (PPh_3_)_2_PdCl_2_ (140 mg, 0.2 mmol) and triphenylphosphine (0.25 g, 0.9 mmol). After the reaction was complete (6 h, TLC analysis), the mixture was diluted with ether (100 mL) and washed with water (50 mL), 5% HCl (50 mL), satd. aq NaHCO_3_ (50 mL), and brined. The organic phase was concentrated under reduced pressure and the residue was purified by chromatography (*n*-hexane/AcOEt 95:5–8:2) and bulb-to-bulb distillation to afford pure (*R*)-γ-cyclogeraniol acetate (−)-**15** = (*R*)-(2,2-dimethyl-6-methylenecyclohexyl)methyl acetate (0.81 g, 78% yield) as a colorless oil; [α]^20^_D_ = −10.1 (*c* 3.8, CHCl_3_); 96% diastereoisomeric purity, 99% of chemical purity by GC. ^1^H NMR (400 MHz, CDCl_3_) δ 4.75 (s, 1H), 4.53 (s, 1H), 4.19 (dd, J = 11.0, 5.3 Hz, 1H), 4.15 (dd, J = 11.0, 9.2 Hz, 1H), 2.16–2.06 (m, 2H), 2.03–1.92 (m, 1H), 1.94 (s, 3H), 1.54–1.44 (m, 2H), 1.42–1.32 (m, 1H), 1.29–1.20 (m, 1H), 0.91 (s, 3H), 0.80 (s, 3H). ^13^C NMR (100 MHz, CDCl_3_) δ 171.0 (C), 147.0 (C), 109.7 (CH_2_), 62.7 (CH_2_), 52.1 (CH), 37.8 (CH_2_), 34.2 (C), 33.3 (CH_2_), 28.6 (Me), 25.0 (Me), 23.3 (CH_2_), 20.9 (Me). Lit. for ^1^H and ^13^C NMR [38,39]. GC-MS *m*/*z* (rel intensity) 136 ([M − AcOH]^+^, 80), 121 (100), 107 (43), 93 (89), 79 (33), 69 (49), 55 (9), 43 (50).

#### 3.3.4. (*S*)-γ-Cyclogeraniol Acetate (+)-**15**

The reaction sequence described above was repeated using sample of diol (−)-**13**, ([α]^20^_D_ = −26.8 (*c* 2.5, CHCl_3_); 90% ee by chiral GC) to afford pure (*S*)-γ-cyclogeraniol acetate (+)-**15** = (*S*)-(2,2-dimethyl-6-methylenecyclohexyl)methyl acetate (74% yield) as a colorless oil; [α]^20^_D_ = +9.1 (*c* 3.1, CHCl_3_); 96% diastereoisomeric purity, 95% of chemical purity by GC. ^1^H-NMR, ^13^C-NMR and GC-MS superimposable to those described for (*R*)-isomer.

### 3.4. Synthesis of the Enantiomeric Forms of the Cyclogeranylsulfonylbenzene Derivatives **11a–d**

#### 3.4.1. General Procedure

A solution of *p*-toluenesulfonyl chloride (1.5 g, 7.9 mmol) in CH_2_Cl_2_ (4 mL) was added dropwise to a stirred solution of the suitable cyclogeraniol isomer (0.9 g, 5.8 mmol), DMAP (50 mg, 0.4 mmol) and pyridine (2 mL) in CH_2_Cl_2_ (4 mL). After 4 h, the mixture was diluted with ether (100 mL) and then was washed with 1 M aqueous HCl solution (50 mL), saturated NaHCO_3_ solution (50 mL), and brined. The organic phase was dried (Na_2_SO_4_) and concentrated *in vacuo*. The residue was dissolved in dry DMSO (5 mL) and added dropwise to a suspension of K_2_CO_3_ (3.2 g, 23.1 mmol) and thiophenol (1.7 g, 15.4 mmol) in DMSO (20 mL). The resulting mixture was stirred vigorously at rt (room temperature) until the starting tosylate was no longer detectable by TLC analysis (12 h). The reaction was partitioned between water (150 mL) and ether (100 mL). The aqueous phase was extracted again with ether (100 mL) and the combined organic phases were washed with an aqueous solution of NaOH (10% w/w, 50 mL) and brined, dried (Na_2_SO_4_), and concentrated *in vacuo*. The residue was dissolved in methanol (50 mL) and was treated at 0 °C with (NH_4_)_2_MoO_4_ (80 mg, 0.4 mmol) followed by the dropwise addition of a solution of H_2_O_2_ (35% wt. in water, 10 mL). The solution was then warmed to rt while stirring was continued for a further 8 h. The reaction was cooled again and a saturated solution of Na_2_SO_3_ was added to destroy excess oxidant. The main part of the methanol was removed under reduced pressure and the residue extracted with AcOEt (3 × 100 mL). The combined organic layers were dried (Na_2_SO_4_), concentrated, and the residue purified by chromatography using *n*-hexane/AcOEt (95:5–8:2) as eluent to afford the suitable cyclogeranylsulfonylbenzene derivative.

#### 3.4.2. (*S*)-((2,6,6-Trimethylcyclohex-2-enyl)methylsulfonyl)benzene (−)-**11a**

According to the general procedure, (*S*)-α-cyclogeraniol (−)-**10a** = (*S*)-(2,6,6-trimethylcyclohex-2-en-1-yl)methanol ([α]^20^_D_ = −102.1 (*c* 2.4, EtOH); 90% ee; 97% chemical purity) was transformed into (*S*)-((2,6,6-trimethylcyclohex-2-enyl)methylsulfonyl)benzene (−)-**11a** (85% yield); [α]^20^_D_ = −83.9 (*c* 2.1, CH_2_Cl_2_); 96% of chemical purity by GC. ^1^H NMR (400 MHz, CDCl_3_) δ 7.97–7.90 (m, 2H), 7.68–7.52 (m, 3H), 5.33 (s, 1H), 3.23 (dd, J = 15.2, 4.3 Hz, 1H), 2.91 (dd, J = 15.2, 3.9 Hz, 1H), 2.25 (s, 1H), 2.06–1.84 (m, 2H), 1.62 (br s, 3H), 1.23–1.16 (m, 2H), 0.91 (s, 6H). ^13^C NMR (100 MHz, CDCl_3_) δ 140.4 (C), 134.8 (C), 133.5 (CH), 129.2 (CH), 128.1 (CH), 121.8 (CH), 58.6 (CH_2_), 42.9 (CH), 32.2 (C), 31.3 (CH_2_), 27.0 (Me), 26.2 (Me), 22.8 (CH_2_), 22.5 (Me). Lit. for ^1^H and ^13^C NMR [25]. MS (ESI): 301.1 [M + Na]^+^.

#### 3.4.3. (*R*)-((2,6,6-Trimethylcyclohex-2-enyl)methylsulfonyl)benzene (+)-**11a**

According to the general procedure, (*R*)-α-cyclogeraniol (+)-**10a** = (*R*)-(2,6,6-trimethylcyclohex-2-en-1-yl)methanol, ([α]^20^_D_ = +96.7 (*c* 2.7, EtOH); 85% ee; 97% chemical purity) was transformed into (*R*)-((2,6,6-trimethylcyclohex-2-enyl)methylsulfonyl)benzene (+)-**11a** (81% yield), [α]^20^_D_ = +77.8 (c 2.8, CH_2_Cl_2_), 98% of chemical purity by GC. ^1^H-, ^13^C-NMR and MS superimposable to those described for (*S*)-isomer.

#### 3.4.4. 2,6,6-((Trimethylcyclohex-1-enyl)methylsulfonyl)benzene **11b**

According to the general procedure, β-cyclogeraniol = ((2,6,6-trimethylcyclohex-1-en-1-yl)methanol (96% chemical purity) was transformed into ((2,6,6-trimethylcyclohex-1-enyl)methylsulfonyl)benzene **11b** (82% yield) as a colorless oil, 97% of chemical purity by GC. ^1^H NMR (400 MHz, CDCl_3_) δ 7.97–7.90 (m, 2H), 7.67–7.51 (m, 3H), 3.97 (s, 2H), 2.06 (t, J = 6.3 Hz, 2H), 1.68–1.58 (m, 2H), 1.67 (s, 3H), 1.52–1.46 (m, 2H), 1.04 (s, 6H). ^13^C NMR (100 MHz, CDCl_3_) δ 141.7 (C), 139.2 (C), 133.2 (CH), 129.1 (CH), 127.8 (CH), 125.9 (C), 57.7 (CH_2_), 39.5 (CH_2_), 34.4 (C), 33.3 (CH_2_), 28.8 (Me), 21.8 (Me), 18.9 (CH_2_). Lit. for ^1^H and ^13^C NMR [40]. GC-MS *m*/*z* (rel intensity) 278 (M^+^, 1), 137 (100), 121 (10), 107 (6), 95 (45), 81 (28), 69 (8), 55 (6).

#### 3.4.5. (*R*)-((2,2-Dimethyl-6-methylenecyclohexyl)methylsulfonyl)benzene (−)-**11c**

(−)-γ-3,4-dehydrocyclogeraniol acetate = (*R*)-(6,6-dimethyl-2-methylenecyclohex-3-en-1-yl)methyl acetate ([α]^20^_D_ = −69.9 (c 3.3, CHCl_3_); 98% ee; 99% chemical purity) was hydrolyzed using NaOH in methanol, at reflux. After work-up, the obtained crude alcohol was submitted to the general procedure to give (*R*)-((6,6-dimethyl-2-methylenecyclohex-3-enyl)methylsulfonyl)benzene (75% yield) as a colorless oil; [α]^20^_D_ = −89.2 (c 3.7, CHCl_3_); 96% of chemical purity by GC. ^1^H NMR (400 MHz, CDCl_3_) δ 7.92–7.86 (m, 2H), 7.66–7.60 (m, 1H), 7.58–7.51 (m, 2H), 5.96 (d, J = 9.9 Hz, 1H), 5.65–5.58 (m, 1H), 4.82 (s, 2H), 3.22 (dd, J = 14.6, 2.4 Hz, 1H), 3.04 (dd, J = 14.6, 8.1 Hz, 1H), 2.53 (dm, J = 8.1 Hz, 1H), 1.90 (dm, J = 18.7 Hz, 1H), 1.79 (dd, J = 18.7, 5.0 Hz, 1H), 0.91 (s, 3H), 0.85 (s, 3H). ^13^C NMR (100 MHz, CDCl_3_) δ 143.1 (C), 140.3 (C), 133.4 (CH), 129.1 (CH), 128.1 (CH), 127.4 (CH), 127.1 (CH), 115.0 (CH_2_), 56.7 (CH_2_), 44.8 (CH), 36.3 (CH_2_), 32.7 (C), 27.2 (Me), 26.7 (Me). MS (ESI): 299.1 [M + Na]^+^, 315.1 [M + K]^+^.

#### 3.4.6. (*S*)-((2,2-Dimethyl-6-methylenecyclohexyl)methylsulfonyl)benzene (+)-**11c**

The reaction sequence described above was repeated using (+)-γ-3,4-dehydrocyclogeraniol acetate = (*S*)-(6,6-dimethyl-2-methylenecyclohex-3-en-1-yl)methyl acetate ([α]^20^_D_ = +61.1 (*c* 3.0, CHCl_3_); 96% of chemical purity) to afford (*S*)-((6,6-dimethyl-2-methylenecyclohex-3-enyl)methylsulfonyl)benzene (74% yield), colorless oil; [α]^20^_D_ = +78.8 (c 3.1, CHCl_3_); 96% of chemical purity by GC. ^1^H-NMR, ^13^C-NMR and GC-MS superimposable to those described for (*R*)-isomer.

#### 3.4.7. (*R*)-((2,2-Dimethyl-6-methylenecyclohexyl)methylsulfonyl)benzene (−)-**11d**

(−)-γ-Cyclogeraniol acetate = (*R*)-(2,2-dimethyl-6-methylenecyclohexyl)methyl acetate (([α]^20^_D_ = −10.1 (c 3.8, CHCl_3_); 96% diastereoisomeric purity; 99% chemical purity by GC) was hydrolysed using NaOH in methanol, at reflux. After work-up, the obtained crude alcohol was submitted to the general procedure to give (−)-(*R*)-((2,2-dimethyl-6-methylenecyclohexyl(methylsulfonyl)benzene (89% yield) as a colorless oil; [α]^20^_D_ = −11.7 (c 3.7, CHCl_3_); 96% diastereoisomeric purity, 95% of chemical purity by GC. ^1^H NMR (400 MHz, CDCl_3_) δ 7.92–7.85 (m, 2H), 7.67–7.49 (m, 3H), 4.74 (s, 1H), 4.56 (s, 1H), 3.35 (dd, J = 14.7, 9.4 Hz, 1H), 3.24 (dd, J = 14.7, 2.5 Hz, 1H), 2.44 (dm, J = 9.4 Hz, 1H), 2.07–1.93 (m, 2H), 1.55–1.44 (m, 2H), 1.35–1.28 (m, 2H), 0.90 (s, 3H), 0.78 (s, 3H). ^13^C NMR (100 MHz, CDCl_3_) δ 145.6 (C), 140.0 (C), 133.4 (CH), 129.0 (CH), 128.2 (CH), 110.9 (CH_2_), 54.4 (CH_2_), 47.8 (CH), 37.2 (CH_2_), 35.3 (C), 32.9 (CH_2_), 27.8 (Me), 24.9 (Me), 23.2 (CH_2_). Lit. for ^1^H and ^13^C NMR [41]. MS (ESI): 301.2 [M + Na]^+^.

#### 3.4.8. (*S*)-((2,2-Dimethyl-6-methylenecyclohexyl)methylsulfonyl)benzene (+)-**11d**

The reaction sequence described above was repeated using (+)-γ-cyclogeraniol acetate = (*S*)-(2,2-dimethyl-6-methylenecyclohexyl)methyl acetate ([α]^20^_D_ = +9.1 (*c* 3.1, CHCl_3_); 96% diastereoisomeric purity, 95% of chemical purity by GC) to afford (*S*)-((2,2-dimethyl-6-methylenecyclohexyl)methylsulfonyl)benzene (84% yield); ([α]^20^_D_ = +9.8 (c 2.4, CHCl_3_); 97% of chemical purity by GC. ^1^H-NMR, ^13^C-NMR and GC-MS superimposable to those described for (*R*)-isomer.

### 3.5. Synthesis of the Furanosesquiterpenes (−)-Pallescensin-1 (**2**), Isomicrocionin-3 (**3**), (−)-Pallescensin-2 (**4**), and (−)-Dihydropallescensis-2 (**5**)

#### 3.5.1. General Procedure

*n*BuLi (1 mL of a 2.5 M solution in hexane) was added dropwise under nitrogen to a cooled (−60 °C) solution of the suitable cyclogeranylsulfonylbenzene derivative (2.2 mmol) in dry THF (10 mL). The resulting orange solution was stirred at this temperature for 15 min and then a solution of 3-(chloromethyl)furan (260 mg, 2.23 mmol) in dry DMPU (1 mL) was added dropwise. The reaction was allowed to reach room temperature and after two hours at this temperature, and it was quenched by the addition of a saturated solution of NH_4_Cl aqueous (50 mL). The resulting mixture was extracted with diethyl ether (3 × 50 mL) and the combined organic phases was dried (Na_2_SO_4_) and concentrated *in vacuo*. The crude product was dissolved in dry THF (15 mL) containing dry Et_2_NH (1 mL). The mixture was cooled (−75 °C) and was treated under nitrogen with freshly prepared lithium naphthalenide (8 mL of a 0.72 M solution). When the staring material could no longer be detected by TLC analysis (1 h), the reaction was quenched by the addition of a saturated solution of NH_4_Cl aqueous (50 mL) and diluted with diethyl ether (80 mL). The organic phase was separated and the aqueous phase was extracted with ethyl ether (50 mL). The combined organic layers were washed with brine, dried (Na_2_SO_4_) and concentrated under reduced pressure. A large part of the naphthalene content was removed by crystallization from hexane. The liquid phase was then purified by chromatography (hexane/diethyl ether 99:1–95:5) to afford the desired furanosesquiterpene.

#### 3.5.2. Synthesis of (−)-Pallescensin-1 (**2**)

According to the general procedure, the alkylation/reduction of ((2,6,6-trimethylcyclohex-2-enyl)methylsulfonyl)benzene ([α]^20^_D_ = −83.9 (c 2.1, CH_2_Cl_2_); 90% ee, 96% of chemical purity by GC) afforded (−)-pallescensin-1 (**2**) = 3-(2-(2,6,6-trimethylcyclohex-2-enyl)ethyl)furan (73% yield) as a colorless oil; [α]^20^_D_ = −93.2 (c 3.2, CHCl_3_); 94% of chemical purity by GC; Lit. [10] for synthetic **2**: [α]^20^_D_ = −89.5 (CHCl_3_); Lit. [3] for natural **2**: [α]^20^_D_ = −23.5 (c 0.07, CHCl_3_). ^1^H NMR (400 MHz, CDCl_3_) δ 7.34 (t, J = 1.7 Hz, 1H), 7.23–7.20 (m, 1H), 6.27 (br s, 1H), 5.31 (br s, 1H), 2.50–2.40 (m, 2H), 2.01–1.91 (m, 2H), 1.76–1.63 (m, 1H), 1.69–1.66 (m, 3H), 1.62–1.39 (m, 3H), 1.19–1.10 (m, 1H), 0.95 (s, 3H), 0.88 (s, 3H). ^13^C NMR (100 MHz, CDCl_3_) δ 142.6 (CH), 138.6 (CH), 136.5 (C), 125.6 (C), 120.2 (CH), 111.0 (CH), 49.0 (CH), 32.6 (C), 31.6 (CH_2_), 31.6 (CH_2_), 27.52 (Me), 27.46 (Me), 25.5 (CH_2_), 23.5 (Me), 23.0 (CH_2_). Lit. for ^1^H and ^13^C NMR [3]. GC-MS *m*/*z* (rel intensity) 218 (M^+^, 8), 203 (2), 162 (3), 147 (15), 133 (13), 121 (14), 109 (26), 95 (41), 81 (100), 67 (7), 53 (13).

#### 3.5.3. Synthesis of Isomicrocionin-3 (**3**)

According to the general procedure, the alkylation/reduction of ((2,6,6-trimethylcyclohex-1-enyl)methylsulfonyl)benzene (97% of chemical purity by GC) afforded isomicrocionin-3 = 3-(2-(2,6,6-trimethylcyclohex-1-enyl)ethyl)furan **3** (78% yield) as a colorless oil; 99% of chemical purity by GC. ^1^H NMR (400 MHz, CDCl_3_) δ 7.34 (t, J = 1.7 Hz, 1H), 7.24–7.22 (m, 1H), 6.30 (br s, 1H), 2.50–2.42 (m, 2H), 2.27–2.19 (m, 2H), 1.93 (t, J = 6.2 Hz, 2H), 1.64 (s, 3H), 1.63–1.55 (m, 2H), 1.47–1.41 (m, 2H), 1.02 (s, 6H). ^13^C NMR (100 MHz, CDCl_3_) δ 142.6 (CH), 138.4 (CH), 136.9 (C), 127.6 (C), 125.7 (C), 110.9 (CH), 39.8 (CH_2_), 34.9 (C), 32.8 (CH_2_), 29.5 (CH_2_), 28.6 (Me), 25.6 (CH_2_), 19.8 (Me), 19.5 (CH_2_). Lit. for ^1^H and ^13^C NMR [3]. GC-MS *m*/*z* (rel intensity) 218 (M^+^, 64), 203 ([M − Me]^+^, 47), 185 (4), 175 (10), 162 (18), 149 (25) 137 (77), 121 (18), 109 (20), 95 (100), 81 (85), 67 (15), 53 (16).

#### 3.5.4. Synthesis of (−)-Pallescensin-2 (**4**)

According to the general procedure, the alkylation/reduction of (*R*)-((6,6-dimethyl-2-methylenecyclohex-3-enyl)methylsulfonyl)benzene ([α]^20^_D_ = −89.2 (*c* 3.7, CHCl_3_); 96% of chemical purity by GC) afforded (−)-pallescensin-2 (**4**) = (*R*)-3-(2-(6,6-dimethyl-2-methylenecyclohex-3-enyl)ethyl)furan (79% yield) as a colorless oil; [α]^20^_D_ = −65.1 (*c* 2.9, CHCl_3_); 96% of chemical purity by GC; Lit. [1] for natural **4**: [α]^20^_D_ = +39.5. 

^1^H NMR (400 MHz, CDCl_3_) δ 7.33 (m, 1H), 7.19 (s, 1H), 6.25 (m, 1H), 6.03 (dd, J = 9.8, 1.8 Hz, 1H), 5.69–5.59 (m, 1H), 4.91 (s, 1H), 4.74 (s, 1H), 2.53–2.42 (m, 1H), 2.33–2.22 (m, 1H), 2.05 (d, J = 18.5 Hz, 1H), 1.81–1.67 (m, 3H), 1.35–1.23 (m, 1H), 0.96 (s, 3H), 0.86 (s, 3H). ^13^C NMR (100 MHz, CDCl_3_) δ 145.6 (C), 142.6 (CH), 138.8 (CH), 127.8 (CH), 127.3 (CH), 125.4 (C), 112.8 (CH_2_), 111.0 (CH), 51.1 (CH), 36.4 (CH_2_), 32.6 (C), 28.3 (CH_2_), 28.2 (Me), 27.6 (Me), 23.0 (CH_2_). Lit. for ^1^H NMR [13]. GC-MS *m*/*z* (rel intensity) 216 (M^+^, 8), 201 ([M − Me]^+^, 2), 173 (2), 157 (2), 145 (2), 131 (2), 122 (55), 107 (100), 91 (17), 81 (10), 65 (4), 53 (5).

#### 3.5.5. Synthesis of (−)-Dihydropallescensis-2 (**5**)

According to the general procedure, the alkylation/reduction of (−)-(*R*)-((2,2-dimethyl-6-methylenecyclohexyl)methylsulfonyl)benzene ([α]^20^_D_ = −11.7 (*c* 3.7, CHCl_3_); 95% of chemical purity by GC) afforded (−)-dihydropallescensis-2 (**5**) = (*R*)-3-(2-(2,2-dimethyl-6-methylenecyclohexyl)ethyl)furan (71% yield) as a colorless oil; [α]^20^_D_ = −7.1 (*c* 2.5, CHCl_3_); 94% of chemical purity by GC; Lit. [15] for synthetic dextrorotatory isomer: [α]^20^_D_ = +4.55 (c 0.1, CHCl_3_); Lit. [2] for natural **5**: [α]^20^_D_ = +6.0 (c 0.3, CHCl_3_). ^1^H NMR (400 MHz, CDCl_3_) δ 7.34 (m, 1H), 7.19 (s, 1H), 6.26 (m, 1H), 4.80 (s, 1H), 4.57 (d, J = 1.9 Hz, 1H), 2.45–2.34 (m, 1H), 2.25–1.92 (m, 3H), 1.81–1.40 (m, 6H), 1.30–1.17 (m, 1H), 0.91 (s, 3H), 0.84 (s, 3H). ^13^C NMR (100 MHz, CDCl_3_) δ 149.1 (C), 142.6 (CH), 138.7 (CH), 125.5 (C), 111.0 (CH), 109.1 (CH_2_), 53.5 (CH), 36.2 (CH_2_), 34.8 (C), 32.4 (CH_2_), 28.3 (Me), 26.7 (CH_2_), 26.3 (Me), 23.7 (CH_2_), 23.3 (CH_2_). Lit. for ^1^H and ^13^C NMR [15]. GC-MS *m*/*z* (rel intensity) 218 (M^+^, 72), 203 ([M − Me]^+^, 14), 189 (6), 175 (10), 162 (5), 147 (12) 133 (13), 124 (13), 109 (85), 95 (65), 81 (100), 69 (49), 53 (21).

### 3.6. Synthesis of (−)-Pallescensone (**6**) Starting from (R)-γ-Cyclogeraniol Acetate

#### 3.6.1. Synthesis of (*R*)-2-(2,2-Dimethyl-6-methylenecyclohexyl)acetonitrile (+)-**16**

(−)-γ-Cyclogeraniol acetate = (*R*)-(2,2-dimethyl-6-methylenecyclohexyl)methyl acetate 380 mg, 1.94 mmol, ([α]^20^_D_ = −10.1 (*c* 3.8, CHCl_3_); 98% ee; 99% chemical purity) was hydrolysed using NaOH in methanol, at reflux. After work-up, a solution of *p*-toluenesulphonyl chloride (570 mg, 3 mmol) in CH_2_Cl_2_ (3 mL) was added dropwise to a stirred solution of the obtained crude alcohol and DMAP (20 mg, 0.16 mmol) in pyridine (2 mL). After 4 h, the mixture was diluted with ether (60 mL) and washed in turn with a 1 M aqueous HCl solution (50 mL), saturated NaHCO_3_ solution (30 mL), and brined. The organic phase was dried (Na_2_SO_4_) and concentrated *in vacuo*. The residue was dissolved in dry DMSO (20 mL) and treated with NaCN (1 g, 20 mmol) stirring at 80–90 °C until the starting tosylate could no longer be detected by TLC analysis (5 h). The mixture was diluted with ether (80 mL) and was washed in turn with water and brine. The organic phase was dried (Na_2_SO_4_) and concentrated *in vacuo*. The residue was then purified by chromatography eluting with hexane/ethyl acetate (95:5–8:2) as eluent to afford pure (*R*)-2-(2,2-dimethyl-6-methylenecyclohexyl)acetonitrile (+)-**16** (270 mg, 85% yield) as a colorless oil; [α]^20^_D_ = +12.1 (*c* 2.2, CH_2_Cl_2_); 96% diastereoisomeric purity, 92% of chemical purity by GC. ^1^H NMR (400 MHz, CDCl_3_) δ 4.96 (s, 1H), 4.75 (s, 1H), 2.56 (dd, *J* = 16.7, 4.4 Hz, 1H), 2.41 (dd, *J* = 16.7, 10.8 Hz, 1H), 2.26–2.15 (m, 2H), 2.14–2.02 (m, 1H), 1.70–1.50 (m, 2H), 1.50–1.31 (m, 2H), 1.00 (s, 3H), 0.81 (s, 3H). ^13^C NMR (100 MHz, CDCl_3_) δ 146.2 (C), 119.7 (C), 110.4 (CH_2_), 50.3 (CH), 37.7 (CH_2_), 35.0 (C), 33.5 (CH_2_), 28.6 (Me), 23.5 (Me), 23.2 (CH_2_), 16.4 (CH_2_). GC-MS *m*/*z* (rel intensity) 163 (M^+^, 3), 148 (7), 134 (1), 120 (21), 107 (9), 91 (11), 79 (18), 69 (100), 53 (11).

#### 3.6.2. Synthesis of (−)-Pallescensone (**6**)

DIBAL (0.9 mL of a 25% wt. solution in toluene, 1.34 mmol) was added dropwise under nitrogen to a cooled (−70 °C) solution of nitrile (+)-**16** (200 mg, 1.23 mmol) in dry toluene (10 mL). The resulting solution was stirred at this temperature for half an hour and then was allowed to reach rt. The reaction was quenched by the carefully addition of diluted HCl aqueous (50 mL), followed by extraction with diethyl ether (2 × 50 mL). The combined organic phases were dried (Na_2_SO_4_) and concentrated under reduced pressure to a final volume of about 4 mL. The resulting solution was added under nitrogen to a cooled (−70 °C) solution of 3-furyllithium (10 mL of a 0.25 M solution in THF), which was previously prepared in situ by addition of *n*BuLi (2.5 M solution in hexane) to a solution of 3-bromofuran in dry THF. The resulting mixture was stirred at this temperature for 20 min, then was quenched by adding saturated aqueous NH_4_Cl (20 mL) and was extracted with ether (2 × 60 mL). The combined organic phases were dried (Na_2_SO_4_) and concentrated under reduced pressure. The residue was dissolved in dry DMSO (2 mL) and was added to a stirred solution of IBX (1 g, 3.5 mmol) in dry DMSO (6 mL). The reaction was warmed at 40 °C for 4 h and then was diluted with diethyl ether (80 mL) and washed with water (2 × 50 mL) and brine. The organic phase was concentrated *in vacuo* and the residue was then purified by chromatography eluting with hexane/ethyl acetate (99:1–9:1) as eluent to afford pure (−)-pallescensone **6** = (*R*)-2-(2,2-dimethyl-6-methylenecyclohexyl)-1-(furan-3-yl)ethan-1-one (145 mg, 51% yield) as a pale yellow oil that solidified on standing; [α]^20^_D_ = −34.4 (*c* 1.1, CH_2_Cl_2_); 97% of chemical purity by GC; Lit. [18] for synthetic **6**: [α]^20^_D_ = −31.8 (c 0.57, CH_2_Cl_2_); Lit. [8] for natural **6**: [α]^20^_D_ = +36 (c 1, CHCl_3_). ^1^H NMR (400 MHz, CDCl_3_) δ 8.05 (s, 1H), 7.44–7.42 (m, 1H), 6.77–6.75 (s, 1H), 4.71 (s, 1H), 4.44 (s, 1H), 2.91 (dd, *J* = 15.8, 9.8 Hz, 1H), 2.81 (dd, *J* = 15.8, 4.2 Hz, 1H), 2.66 (dd, *J* = 9.8, 4.2 Hz, 1H), 2.28–2.17 (m, 1H), 2.13–2.01 (m, 1H), 1.65–1.34 (m, 4H), 0.98 (s, 3H), 0.84 (s, 3H). ^13^C NMR (100 MHz, CDCl_3_) δ 194.5 (C), 148.9 (C), 146.7 (CH), 144.1 (CH), 128.1 (C), 108.8 (CH), 108.5 (CH_2_), 48.7 (CH), 38.8 (CH_2_), 38.6 (CH_2_), 35.0 (C), 34.4 (CH_2_), 28.9 (Me), 23.8 (Me), 23.6 (CH_2_). Lit. for ^1^H and ^13^C NMR [14,16,18]. GC-MS *m*/*z* (rel intensity) 232 (M^+^, 5), 217 (5), 199 (2), 189 (3), 176 (3), 163 (3), 137 (3), 122 (22), 107 (24), 95 (100), 81 (12), 69 (13), 55 (6).
